# Cyanobacteria provide a new paradigm in the regulation of cofactor dependence

**DOI:** 10.1073/pnas.2100281118

**Published:** 2021-02-05

**Authors:** Crysten E. Blaby-Haas

**Affiliations:** ^a^Biology Department, Brookhaven National Laboratory, Upton, NY 11973;; ^b^Department of Biochemistry and Cell Biology, Stony Brook University, Stony Brook, NY 11794

Competition for resources is a decisive driver for success. Entire civilizations have collapsed when food scarcity leads to conflict. Competition for nutrients has also shaped evolution, with modern-day species descending from the victors. While archeological digs and historical accounts yield insight into how resource competition impacted human history, whole-genome sequencing is needed to understand the inherited adaptations that passed the filter of natural selection. Leveraging comparative genomics, García-Cañas et al. (1) report their discovery of how cyanobacteria won the arms race when confronted with resource limitation caused by copper deficiency. Cyanobacteria are an ancient group of bacteria credited with the evolution of oxygen-producing photosynthesis, a process with an absolute requirement for metal cofactors, including iron, manganese, and copper. García-Cañas et al. (1) find that a BlaI-family transcription factor and BlaR1-family protease evolved to regulate the transcription-mediated swap of copper-dependent plastocyanin (PC) for iron-dependent cytochrome *c*_6_ (Cyt*c*_6_) (1). Over 40 y ago, researchers studying green algae (2) and cyanobacteria (3) proposed that replacement of PC with Cyt*c*_6_ provides a strategy to maintain photosynthesis with a “back-up” protein when PC becomes inactivated due to copper insufficiency, but how this interchange is regulated in cyanobacteria remained unknown until now.

PC and Cyt*c*_6_ are functionally identical, small, soluble, electron-transfer proteins that transfer one electron at a time from the cytochrome *b*_6_*f* complex to photosystem I during oxygenic photosynthesis. However, that is where their similarities end. The two proteins are evolutionarily unrelated, differing in both primary sequence and tertiary structure. The most striking difference between these two isofunctional proteins is their cofactors. Cyt*c*_6_ contains a tetrapyrrole-bound iron atom (i.e., heme) that switches between Fe^3+^ and Fe^2+^ to accept and donate an electron. Conversely, PC utilizes a single copper ion that switches between Cu^2+^ and Cu^1+^ to accept and donate an electron. Although aqueous iron and copper ions have dramatically different redox potentials (the standard reduction potential of Fe^3+^ is 0.77 V and of Cu^2+^ is 0.159 V), the metal ions in PC and Cyt*c*_6_ are tuned to roughly 370 mV, the redox potential needed to catalyze the transfer of an electron from cytochrome *f* to P700^+^ in photosystem I (in cyanobacteria and green algae) or to the respiratory terminal oxidase (in cyanobacteria).

In addition to showcasing the role of polypeptides in manipulating the chemical properties of metal cofactors, these two proteins are a remarkable example of convergent evolution driven by access to metals. Based on historical estimates of iron and copper bioavailability, Cyt*c*_6_ is thought to have evolved first. Before oxygen levels in the atmosphere rose due to oxygenic photosynthesis in the early Paleoproterozoic [∼2,400 Ma (4)], iron was plentiful (the fourth-most-abundant element in Earth’s crust) and bioavailable as the water-soluble, reduced form of iron (Fe^2+^). However, as oxygen levels increased, iron became more limited, as oxidation of Fe^2+^ resulted in the insoluble form of iron (Fe^3+^). At the same time, copper that was previously locked in the water-insoluble Cu^1+^ state was oxidized to the more soluble Cu^2+^ state, leading to the evolution of copper-dependent proteins, such as PC.

**Fig. 1.**
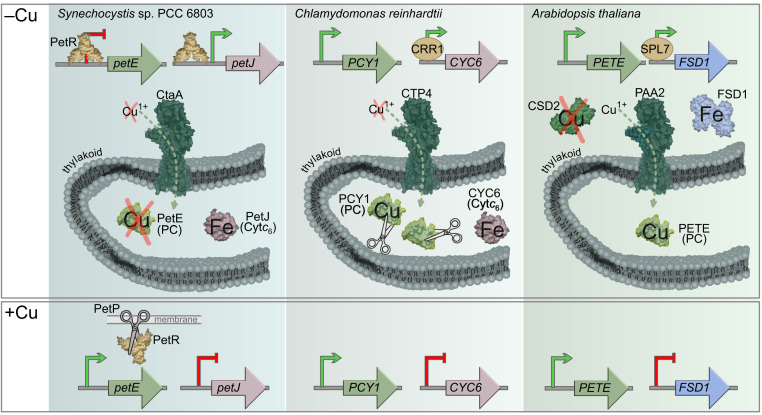
Comparison of PC/Cyt*c*_6_ regulation across oxygenic phototrophs. In cyanobacteria, green algae, and land plants, the functional orthologs CtaA, CTP4, and PAA2, respectively, pump Cu^1+^ into the thylakoid lumen for PC biogenesis (14–16). In cyanobacteria and green algae, where genes encoding both PC and Cytc_6_ are present, copper depletion leads to replacement of PC with Cyt*c*_6_. As described by García-Cañas et al. (1), in *Synechocystis* sp. PCC 6803 transcription is regulated by binding of PetR to the promoters of *petE* and *petJ*. In turn, PetR is regulated posttranslationally by the protease PetP in the presence of copper. By comparison, in *C. reinhardtii* transcription of *PCY1*, encoding PC, is constitutive, while transcription of *CYC6*, encoding Cyt*c*_6_, is activated by CRR1. PC abundance is regulated posttranslationally by an unidentified protease. In land plants, copper distribution to PC is prioritized during copper deficiency in part by the copper-regulated replacement of stroma-localized Cu/Zn superoxide dismutase (CSD2) and Fe-dependent superoxide dismutase (FSD1) (17). The CRR1 functional ortholog SPL7 activates transcription of *FSD1* and an artificial microRNA family (miR398) that simultaneously reduces transcript abundance of *CSD2* (18).

The availability of two isofunctional proteins, one that uses a copper ion and one that uses a heme cofactor, facilitated nutritional flexibility. Some oxygenic phototrophs, such as land plants and a handful of green algae, only contain PC, while others, such as red algae, only contain Cyt*c*_6_. A third group, composed of green algae and cyanobacteria, contains genes for PC and Cyt*c*_6_. The presence of both genes enables these phototrophs to “pick” which protein, and, therefore, which metal cofactor, to use for electron transfer. PC is expressed if copper is plentiful; when copper becomes limiting, PC is replaced with Cyt*c*_6_. As characterized previously in the green alga *Chlamydomonas reinhardtii*, a plant-specific transcription factor, CRR1, activates transcription of the Cyt*c*_6_-encoding gene during copper deficiency (5), expression of the PC-encoding gene is constitutive (6), and an unidentified protease(s) targets both copper-bound PC and metal-free PC for degradation (7, 8) (Fig. 1). Although the outcome is the same, and green algae likely acquired the genes for PC and Cyt*c*_6_ through gene transfer from the cyanobacterium-like progenitor of the chloroplast, García-Cañas et al. find that the regulatory mechanism responsible for the PC/Cyt*c*_6_ exchange is not conserved between cyanobacteria and green algae (1).

Since PC- and Cyt*c*_6_-encoding genes (*petE* and *petJ*, respectively) are regulated by copper at the transcriptional level (9, 10) in cyanobacteria, García-Cañas et al. (1) performed a comparative genomic analysis of available cyanobacterial genomes with a focus on identifying putative transcription factor genes neighboring *petE* or *petJ.* They identified an orthologous group of genes encoding an uncharacterized BlaI-like transcription factor (named PetR) and a neighboring orthologous group of genes encoding an uncharacterized BlaR1-like protease (named PetP). To test the hypothesis that these proteins are responsible for the reciprocal expression of *petE* and *petJ*, García-Cañas et al. (1) leverage *Synechocystic* sp. PCC 6083 as a reference experimental system. Interpretation of *petE* and *petJ* transcript abundance in *petR* and *petP* deletion strains combined with evidence from electrophoretic mobility shift assays leads to the conclusion that PetR is needed to repress the expression of *petE* and activate the expression of *petJ* (Fig. 1). PetP has an opposite effect on expression consistent with the role of this protease in PetR degradation. In the consequential absence of PetR, *petE* expression is no longer repressed and *petJ* expression is no longer induced, phenocopying the *petR* mutant. García-Cañas et al. (1) further propose that PetP has copper-dependent protease activity, providing a mechanism for copper sensing. Coincidently, an unidentified copper-activated protease has also been found to play in role in copper-sensing by *Enterococcus hirae* (11), suggesting that the role of proteases in regulating copper-responsive transcription extends beyond cyanobacteria.

In addition to uncovering a paradigm in copper sensing, the findings of García-Cañas et al. (1) provide the foundation for exciting research avenues. Although dual activator–repressor activity has been described for other transcription factors that regulate metal homeostasis, such as Zur (12), the mechanism enabling opposite outcomes of transcription factor–DNA binding by PetR is unknown. Future work into the copper-regulated activity of PetP promises to reveal a new mechanism for establishing fidelity of metal selectivity in regulatory circuits. Additionally, RNA sequencing of *petP* and *petR* mutant strains reveal that the copper-regulated PetRP regulon only encodes four proteins: PC, Cyt*c*_6_, and two proteins of unknown function (1). Of these two uncharacterized proteins, Slr0602 was previously found to interact with six photosynthetic proteins, including PC (13), suggesting that replacement of PC with Cyt*c*_6_ may involve posttranslational adjustment of some photosynthetic complexes, hinting at potentially uncharted complexity.
